# Scouring in Calves*Read before the Massachusetts Veterinary Association, April, 1893.

**Published:** 1893-10

**Authors:** John M. Parker


### SCOURING IN CALVES.*

By John M. Parker.

Diarrhœal diseases in calves may be roughly divided into simple dyspeptic diarrhœa and " acute mycotic diarrhœa or diarrhœa of bacterial origin."

The difference between the two is rather one of degree than one of kind. In the former the symptoms are not so severe ; it is rarely fatal, and the treatment is practically the same as in the more acute form, which is the form I shall more particularly consider.

Within the last ten years numerous investigators have been at

* Read before the Massachusetts Veterinary Association, April, 1893.

work on the aetiology of diarrheal diseases, but so far no very positive conclusions have been reached. It appears to be generally accepted, however, that diarrheal diseases are due to bacteria or their products.

It is now known that bacteria are present in the normal intestine in enormous quantity, and further that different kinds of food favor the growth of different kinds of bacteria. In infants or animals fed on milk diet, for example, there are two kinds of bacteria that are always present; one of these, the "bacterium lactis aerogenis," is only present during milk diet; "it has not been found in the meconium, nor after a flesh diet, and it is found principally in the upper portion of the intestinal tract." As pointed out by Escherick, "Its vital activity in the intestine depends on the presence of milk sugar, and its extension there corresponds with that of this substance."

Milk sugar is completely absorbed in the stomach and small intestine, and is not found normally in the large intestine. At the time of milk digestion the "bacterium lactis aerogenis" is found in great quantity in the upper part of the small intestine, and in proportion to the absorption of milk sugar it becomes scarce, diminishing in number, in the color, and in the faeces comparatively few individuals of the species are found. (Keating, p. 180.)

Baginsky has demonstrated by experiment that the "bacterium lactis aerogenis" has an extremely active development, and when cultures are made along with other pathogenic bacteria in suitable culture media, the "bacteria lactis aerogenis" grows so rapidly that it prevents and retards the growth of the other bacteria. These experiments led him to the opinion that the "bacterium lactis aerogenis" if placed under favorable conditions may prevent the growth of other pathogenic organisms in the intestine, and that "in the acid fermentation of milk sugar caused by the 'bacterium lactis aerogenis,' we have a remedy which serves in the infant's organism to protect the intestinal wall from pathogenic bacteria." But when this fermentation exceeds a certain degree, which may happen in abnormal conditions of the intestine, it destroys the "bacterium lactis aerogenis," and thus lays the foundation for pathogenic processes of various kinds. (Keating, p. 185.) For example, if from some temporary cause there is increased peristalsis, or an intestinal catarrh, or if from any cause there is an interference with the normal process of digestion, the temporary trouble may be kept up and aggravated by the presence of the micro-organisms which have gained the ascendency through the destruction of the normal bacterium.

These micro-organisms "may have begun their work outside the body by developing in the milk and so causing abnormal products of decomposition or fermentation, possibly poisonous ptomains." These abnormal products of decomposition or poisonous ptomains affect principally the nervous centres and cause the severe nervous depression and heart failure usually seen in these cases.

Baruch also believes "that the summer diarrhea of infants is chiefly, though not solely, due to the ingestion of micro-organisms which cause, in the gastro-intestinal tract, conditions analogous to those found in wounds, to which septic material has had access." He further argues that unsanitary conditions, poverty, filth, etc., are entitled to be placed only among the predisposing causes. The indigestibility of caseine of cow's milk by artificially fed children is of no importance, since in winter this is borne without serious consequences. He concludes that the kind of food, if it be reasonably constructed, has little to do with producing diarrheal disease, *provided the access of micro-organisms in its preparation can be prevented.*

Rachford also holds, 1st—That the chief, if not the only direct, cause of "summer complaint" is abnormal intestinal fermentations of food stuffs. These are always caused by bacteria.

2nd—At present we are unable to make an exact ætiological classification of these diseases, but it is probably true that there are quite a number of pathogenic bacteria, each capable of producing definite changes in the food which will cause characteristic symptoms.

3rd—The disease, being of bacterial origin, is necessarily infectious. It is probable that not all the diseases embraced under the general term are equally infectious.

This writer believes that bacteria may cause disease in any one of three ways:

1st—By interfering with the growth and function of bacteria normal to the intestine. In this way bacteria which do not produce poisonous ptomains or irritating products may cause digestive derangements. It is quite likely that this form of disease may act as a predisposing cause to other forms.

2nd—By formation of irritating materials during the fermentation of food stuffs in the intestine, especially in acid fermentation.

3rd—By producing ptomains which act as physiological poisons. (Universal Med. Sciences, vol. i, 1889.)

All investigators seem unanimously to be of the opinion that bacteria either directly or indirectly are *the* cause of this class of disease. There seems to be a tendency, however, to lay too much stress on the bacterial origin of the disease and forget that diet and constitution, and the sanitary and hygienic surroundings, have a great deal to do with its fatal character.

As showing the influence of diet on the prevalence of diarrhœal diseases in children, out of 1,000 cases recorded by Hope, 30 were fed by the breast exclusively; of 602 fatal cases recorded by Meineot, 24, and of 34 fatal cases recorded by Ballard, 7 were fed by the breast exclusively, making a total of 1943 fatal cases, of which but or about 3 per cent. had the breast exclusively.

"These facts," says Keating, "speak volumes; they show that the manner of feeding is one of the most important factors in the production of diarrhœa; as long as children are nursed exclusively they suffer but little from diarrhœal disease, but in the same class of children as soon as the age is reached when other food is added, we find a very marked increase in its frequency. Children among the poor in tenements enjoy immunity from intestinal disease just in proportion as they are nursed at the breast, and just so long as they are so; but as soon as artificial feeding is begun diarrhœal diseases begin to be prevalent." (Keating, p. 64.)

These remarks apply equally well to calves. In the summer of 1890 and '91 I treated from 45 to 50 cases of scouring in calves. The calves averaged from 1 to 3 months old when attacked. They were usually allowed to suck until 3 or 4 weeks old, when they were hand-fed till 3 or 4 months old, at which time, if the season of the year was suitable, they were turned out in pasture.

With seven exceptions all the cases occurred between the time of weaning and turning out to pasture, and with three exceptions all the cases occurred during the summer months. Six cases occurred while the calf was suckling (morning and night); over forty while the calves were confined in pens and were being hand-fed, and only one after the calves were turned out to pasture. On one occasion calf after calf was attacked with scouring, two or three new cases occurring each day until there were upwards of a dozen calves sick at one time. Three had died, and nothing seemed to do any good until on close investigation we found that great numbers of the squashes, which were being fed the cows, were decayed. Their use was discontinued, and the scouring rapidly disappeared. That this was the cause of the trouble was proved by the fact that some weeks later the foreman, wishing to use up the remainder of the squashes, to get them out of the way, fed them to the cows, with the result that more of the calves were attacked with the old trouble.

Injudicious feeding then of cow or calf, allowing the calf to overgorge itself, irregular feeding, feeding milk too hot or too cold, all unfavorable hygienic conditions, such as hot, close weather, over-crowding, bad ventilation, want of sunlight, want of exercise, want of bedding; any one of these or a combination of them tend to produce an unhealthy condition of the calf and favor chronic indigestion. "And this chronic dyspepsia (or indigestion, says Keating) is more important than all other factors as a predisposing cause of diarrhœal disease." Anything that lowers the vitality of the animal increases its liability to disease. On the other hand, "healthy digestion and perfect absorption" are the great obstacles to the "development of new varieties of bacteria," for, "although new varieties of bacteria are being introduced all the time, they fail to develop, because their number is small or the conditions favorable to their development are wanting."

Suckling calves escape because they are healthier; because their digestive organs have not been abused, and because their milk is sweet and pure and *free from bacteria.* While on the other hand, calves brought up by hand are usually more or less troubled with chronic indigestion from one or other of the causes already mentioned.

The pails and milking utensils and receptacles for milk are not usually so pure and clean as they ought to be, and partly for that reason, and in consequence of the hot weather in summer, the milk often swarms with germ life, and is sour and acid from fermentation having already commenced, so that when taken into the stomach, especially if taken in large quantity, and in hot weather when the system is enervated, and there is a low state of vitality, the digestive power is to a great extent lost, food will ferment and produce irritating acids and ptomains, which in turn produce the symptoms which we know as "Scouring in Calves."

*Symptoms.*—The symptoms of acute mycotic diarrhœa in calves are well marked. The onset is usually sudden. One of the first symptoms noticed is usually the want of appetite. The calf refuses food entirely; the tail and buttocks are noticed to be foul and covered with a dirty, yellowish white discharge; its eyes appear sunken, there is often a discharge from both nose and eyes; the nose is usually dry; it rapidly loses flesh and strength; it lies continually in a semi-comatose condition with its eyes wide open, and it has not even vitality to brush off the flies that are attracted by the sour-smelling evacuation; if made to rise it will stagger and appear weak. The abdominal walls appear flat and collapsed; its extremities are cold; its muscles become flabby; sometimes when it has been lying with its head round to its side, when made to move the muscles of the neck will have contracted so that it is unable to straighten its neck; it keeps continually pressing its teeth against ts gums, causing a peculiar, rubbing noise. The pulse is rapid and weak and hard to count. The temperature is usually elevated.

This disease runs a particularly rapid course, the severe symptoms seldom lasting over 48 hours, and in some cases it reaches a fatal termination in from 8 to 10 hours. Three cases have come under my notice where the calves were seemingly well in the evening and were found dead the following morning.

In simple dyspeptic diarrhea, while the symptoms may sometimes develop almost as rapidly, yet they are not so severe, there is not the sunken appearance of the eyes; the calf does not lie in the same semi-comatose condition; there is not the same evidence of nervous depression, but it appears brighter; it pays more attention to surrounding objects; there is no rise in temperature, and the appetite is not entirely gone.

The prognosis in all diarrhœal diseases should be guarded if the symptoms are severe; if the patient lies in a comatose condition, with no appetite, sunken eyes and profuse diarrhœa; especially if the hygienic conditions are poor, then the prognosis is unfavorable; if, on the contrary, the appetite is not altogether gone, if the eyes are bright and the hygienic conditions and sanitary surroundings are good, the prognosis is much more favorable.

*Post-mortem appearances.*—In making autopsies on calves dying from this disease, one cannot help noticing the marked absence of lesions, when compared with the great severity of the symptoms.

This is fully accounted for, however, when we remember that the process is not inflammatory, but through acute fermentation in the gastro intestinal tract a poisonous substance is produced, which affects primarily the nerve centres, causing nervous depression and heart failure. If this process were continued for a sufficient length of time, inflammatory changes would take place ; as it is death usually occurs before the inflammatory lesions are sufficiently well marked to be noticeable to the naked eye.

In ten post-mortem examinations which I have made in calves dying from "acute mycotic diarrhœa," the examinations were held within a few hours after death, and the appearances observed were in all cases practically the same.

The first thing that strikes one in looking at the body is the rapidity with which emaciation has gone on, and the completely collapsed condition of the abdominal walls. The eyes are sunken, there is discharge from both eyes and nose; the tail is invariably soiled On making an incision the first point to be noticed is the pale, white character of the muscles and intestines; the lungs appear normal except for hypostatic congestion; the heart usually contains a quantity of dark colored blood in both sides; the heart muscles are flabby and soft.

The intestines contain dirty white liquid, consisting of indigested food and particles of curdled milk. In most instances the mucous membrane is soft and easily separated, but it must be remembered that it is usually hot weather and the post-mortem changes are very rapid.

The stomach usually contains a quantity of frothy, undigested food, liquid food, the mucous membrane are all pale in color and have a washed-out appearance.

Other abdominal organs appear normal ; notwithstanding the absence of pathological changes; however, the conditions would not be easily mistaken. The flabby condition of the buttocks, sunken eyes, paleness of intestinal organs, all tell a tale that would enable one to recognize a case, even if there was no history to guide him.

*Treatment.*—Scouring in calves is an exceedingly indefinite term ; it seems to include all cases of simple dyspeptic diarrhœa, acute mycotic diarrhœa, and even the more chronic forms take shelter under its wing ; and in considering the treatment to be adopted, while the general plan of treatment is the same in all, yet the success attending treatment in the more severe forms is usually far from encouraging.

The essential points to be remembered in treating a case of acute mycotic diarrhœa are first and foremost to sustain and strengthen the heart and system generally, to allay nervous irritation and to stop diarrhœal discharges.

The treatment with which I have had the greatest success is to clear out the gastro intestinal tract with laxative medicine (nothing answers the purpose better than Castor Oil, two or three ounces), to stop all milk diet for from twelve to forty-eight hours, and to support the system with brandy and eggs and oatmeal water. The brandy and water should be used freely.

Opium is a good heart tonic, and at the same time it diminishes the excessive hyper-peristaltic movements of the bowels.

Probably Dover's Powder is as good a form as any in which it can be given.

Where there is much stupor Chlorodyal or Specae is preferable to opium. Sometimes suppositories of opium are of great value.

It should always be borne in mind that the simpler the medicinal treatment the better. Laxative medicine clears the intestinal tract of all irritating substances and products of fermentation, allowing it to begin afresh, "so to speak." After from twenty-four to forty-eight hours have elapsed probably the best results are obtained by allowing the calf to suck a little, if it will. If it refuses, then a very little diluted sterilized milk (not more than one half pint at a time) may be given with the brandy and eggs and some preparation of opium.

Medicinal treatment, however, is of secondary importance to dietetic and hygienic treatment; good air, pure air, and plenty of it, is an absolute necessity: nothing is more depressing, especially in summer weather, than for a sick animal to be shut up in a hot, close pen with little light or air; and when we remember that these are cases of "poisoning, with great nervous depression of the heart and system generally, and that we are not treating intestinal catarrh, nor intestinal inflammation, although intestinal inflammation is one of the results that is likely to follow if the patient survives the first overwhelming shock of the poison," the necessity for pure air and good light and clean surroundings is still further emphasized.

During convalescence too much care cannot be taken; if the calf is very young it should be allowed to suck a cow that has lately come in; if that is impossible then it should be fed scalded milk for some time, and the greatest care should be taken with the surrounding hygienic conditions.

Diarrhœal diseases are to a great extent preventable; if "scouring" is due to the introduction of micro-organisms, and if conditions favoring the growth of micro-organisms favor the development of the disease, then it follows as a natural consequence that the removal of these conditions will be followed by a corresponding decrease in the prevalence of the disease. It is most important, then, that the milk should be kept in as cool a place as possible, the utensils ought to be scalded both before and after using (in consequence of the difficulty in keeping them clean, wooden pails should not be used).

The milker's hands and the udder should both be washed before milking, and in every way an endeavor should be made to keep the milk fresh and sweet, and *free from bacteria.*

Further, the calves should be kept in as strong and healthy a condition as possible; the pens in which they are kept should have good light and plenty of fresh air; these are just as essential to the healthy development of animal life as of plant life. Dry bedding and plenty of it is another item that is too often neglected. The calf pens are usually in a dirty, filthy condition; on the average farm they are not cleaned out till a wet day comes round, when the hired man does odd jobs around the buildings. Nothing is worse for young stock of any kind than to be compelled to lie in their own wet and filth.

Another important matter is the water supply. Calves will drink a large quantity of water if they have free access to it; but when the wells are situated in or near the barn yard, more or less of the surface drainage must find its way into the well and contaminate the water supply.

In conclusion, the whole sum and substance of prevention is *hygienic.* Hygienic is of paramount importance; unfortunately, however, farmers do not realize the importance of the subject, and until they do, "Scouring in Calves" will remain as much of a mystery and a source of loss to the farmer as it is to-day.

